# Detection and Investigation of Extracellular Vesicles in Serum and Urine Supernatant of Prostate Cancer Patients

**DOI:** 10.3390/diagnostics11030466

**Published:** 2021-03-08

**Authors:** Samanta Salvi, Erika Bandini, Silvia Carloni, Valentina Casadio, Michela Battistelli, Sara Salucci, Ilaria Erani, Emanuela Scarpi, Roberta Gunelli, Giacomo Cicchetti, Michele Guescini, Massimiliano Bonafè, Francesco Fabbri

**Affiliations:** 1 Biosciences Laboratory, Istituto Scientifico Romagnolo per lo Studio e la Cura dei Tumori (IRST) IRCCS, Via P. Maroncelli 40, 47014 Meldola, Italy; erika.bandini@irst.emr.it (E.B.); silvia.carloni@irst.emr.it (S.C.); valentinacasadio.bionut@gmail.com (V.C.); ilariaerani@gmail.com (I.E.); francesco.fabbri@irst.emr.it (F.F.); 2 Department of Biomolecular Sciences, University of Urbino Carlo Bo, 61029 Urbino, Italy; michela.battistelli@uniurb.it (M.B.); sara.salucci@uniurb.it (S.S.); michele.guescini@uniurb.it (M.G.); 3 Unit of Biostatistics and Clinical Trials, Istituto Scientifico Romagnolo per lo Studio e la Cura dei Tumori (IRST) IRCCS, Via P. Maroncelli 40, 47014 Meldola, Italy; emanuela.scarpi@irst.emr.it; 4 Department of Urology, Morgagni Pierantoni Hospital, 47121 Forlì, Italy; gunellifiori@gmail.com; 5 Department of Urology, Bufalini Hospital, 47521 Cesena, Italy; gcicchetti@hotmail.com; 6 Department of Experimental, Diagnostic and Specialty Medicine, AlmaMater Studiorum, University of Bologna, 40126 Bologna, Italy; massimiliano.bonafe@unibo.it

**Keywords:** prostate cancer, extracellular vesicles, urine, serum, MACSPlex Exosome kit

## Abstract

Prostate Cancer (PCa) is one of the most frequently identified urological cancers. PCa patients are often over-diagnosed due to still not highly specific diagnostic methods. The need for more accurate diagnostic tools to prevent overestimated diagnosis and unnecessary treatment of patients with non-malignant conditions is clear, and new markers and methods are strongly desirable. Extracellular vesicles (EVs) hold great promises as liquid biopsy-based markers. Despite the biological and technical issues present in their detection and study, these particles can be found highly abundantly in the biofluid and encompass a wealth of macromolecules that have been reported to be related to many physiological and pathological processes, including cancer onset, metastasis spreading, and treatment resistance. The present study aims to perform a technical feasibility study to develop a new workflow for investigating EVs from several biological sources. Serum and urinary supernatant EVs of PCa, benign prostatic hyperplasia (BPH) patients, and healthy donors were isolated and investigated by a fast, easily performable, and cost-effective cytofluorimetric approach for a multiplex detection of 37 EV-antigens. We also observed significant alterations in serum and urinary supernatant EVs potentially related to BPH and PCa, suggesting a potential clinical application of this workflow.

## 1. Introduction

Prostate Cancer (PCa) is the most frequently diagnosed non-cutaneous urological cancer and the second main cause of cancer-related death affecting men, with an estimated amount of 191,930 new cases and 33,330 predicted deaths in the US in 2020 [1]. As for other types of tumors, the lack of an early manifestation represents a major issue in the management of PCa, mainly monitored through prostate specific antigen (PSA) in blood in the early stages. In addition, benign prostatic hyperplasia (BPH) is a non-malignant enlargement of the prostate due to a cellular growth that develops within the transitional zone [2], which can be associated with elevated levels of PSA, as well as in prostatitis. The low specificity of PSA tests results in innumerable useless biopsies: only 25% of patients are found to have PCa in the following biopsy using the current established threshold of 4 ng/mL [3]. Particularly, the approved PSA tests result in failure of the discrimination between benign hypertrophy and cancer with the consequence that patients undergo more painful and invasive procedures [4]. Liquid biopsy (LB) is today explored in the context of circulating subcellular components and the investigation of biological fluids is gaining great attention because of some features common to the tissue of origin, especially for the more easily accessible fluids such as blood (plasma or serum) and urine. In particular, urine has become one of the most interesting bio-fluids in clinical practice due to its easy collection method, its availability in terms of quantities, and its non-invasiveness. Furthermore, compared to blood, urine is a less complex and relatively clean biofluid, with the only relatively abundant protein being uromodulin [5]. Besides cell-free DNA, circulating tumor cells (CTCs), circulating RNAs (miRNA, lncRNAs and mRNAs), proteins and peptides, and extracellular vesicles (EVs) are becoming a promise in the context of LB [6]. EVs are lipid-enclosed particles containing several macromolecules (cargo) that depend on the cell of origin. EVs are highly abundant (~1–3 × 10^12^ per mL of plasma/serum) and closely related to biological characterization of the tumor [7,8]. In particular, exosomes belong to the category of small EVs (from 30 to 120 nm in diameter) [9] and they generally contain molecules as miRNAs, proteins (e.g., tetraspanin CD63, CD81, CD82, CD53 and CD37), lipids (e.g., sphingomyelin, cholesterol and saturated fats), and viral particles [10]. In general, PCa-associated exosomes were found to be characterized by a cargo containing cancer-related proteins such as CD9, CD81, and TSG101, Annexin A2, Fatty Acid Synthase (FASN), and prostate-specific membrane antigen (PSMA) [11]. The challenges in developing ideal early detection markers for PCa are outstanding, and numerous new blood-based and urinary biomarker models are emerging for usage in PCa detection, follow-up and treatment decision-making, despite current methods having faced complications with efficiently isolating EVs from biofluids [12,13]. One of the most tempting challenges would be that of better analyzing the exosome’s surface, as some groups recently reported in their studies using different approaches [14], and to deeply investigate more reliable isolation and analysis methods, focusing on a subset of circulating exosomes enriched for tumor origin, rather than total serum/plasma or urine exosomes [15]. The plethora of methods to separate EVs from biofluids highlights major concerns such as material of extremely variable purity and missing data referable to technical repeatability, which put an obstacle to clinical translation. Despite their great potential to improve patient care, there are a number of biological, technical, and clinical questions that need to be addressed before LB can be adopted into clinical practice, and a crucial point is to explore where EVs come from, whether they arise from the primary tumor site or metastatic lesions. The aim of the present study was to develop a technical feasibility study based on a new workflow to investigate the potential role of EVs. EVs from serum and urinary supernatants of PCa and BPH patients and healthy donors were isolated and then phenotyped by a bead-based cytofluorimetric approach able to detect 37 surface exosomal-related proteins.

## 2. Materials and Methods

### 2.1. Prostate Cancer Case Series

The study was conducted on 30 individuals: 10 PCa patients, 10 individuals with BPH, and 10 healthy donors (H). The samples were enrolled between 2013 and 2014 at the Morgagni and Pierantoni Hospital (Forlì, Italy) and Bufalini Hospital (Cesena, Italy). All PCa patients underwent radical prostatectomy. The Gleason score and pathological stage were evaluated after surgical removal of the tumor. Healthy donors were matched to PCa and PBH for age classes (<70 and ≥70 years). Available clinical data are reported in Supplementary Table S1. This study was approved by the Local Ethics Committee (Comitato Etico Area Vasta Romagna and IRST) and informed consent was obtained from all patients (protocol number: L3P21, approved in 2013 and updated in 2019).

### 2.2. Serum and Urine Collection

The blood and the first-morning voided urine samples were collected from all individuals before any surgical intervention. 

Approximately 5 mL of whole blood were collected in a serum tube without anticoagulant and centrifuged at 1000× *g* for 15 min at 4 °C for obtaining serum. 

The urine samples were maintained at 4° C for a maximum of 3 h until processing. Approximately 30 mL of urine were aliquoted and centrifuged at 850× *g* for 10 min. The urinary supernatant was collected into cryovials.

### 2.3. EV Isolation and Quantification

EVs from serum and urinary supernatants were isolated using Total Exosome Isolation (TEI) from serum reagent (Thermo Fisher Scientific, Inc., Waltham, MA, USA) and TEI from urine reagent (Thermo Fisher Scientific Inc.), respectively. Five hundred µL of serum and 2 mL of urinary supernatant were used. Briefly, 100 µL and 2 mL of the specific TEI reagent were added to the serum and urinary supernatant, respectively. After incubation and centrifugation according to the manufacturer’s protocol, the EVs pellet obtained from serum or urinary supernatant was resuspended in 300 µL or 250 µL of 0.22 µm-filtered 1× PBS, respectively. The EVs quality and quantity were checked using Nanosight NS300 (Malvern Panalytical, Worcestershire, UK), performing sample dilution to obtain an optimal particle/frame result.

### 2.4. TEM Analysis

EVs isolated from serum and urinary supernatant were adsorbed to formvar carbon-coated 200 mesh grids (Agar Scientific Ltd., Stansted, UK) for 20 min. After the grids were dried, they were incubated with 2% (*w*/*v*) sodium phosphotungstate for 1 min and the liquid excess was removed with filter paper. After negative staining, specimens were observed by means of a Philips CM10 transmission electron microscope at 80 kV [16].

### 2.5. MACSPlex Analysis

The MACSPlex Exosome Kit (Miltenyi Biotec, Bergisch Gladbach, Germany) allows the detection of 37 exosomal surface epitopes (CD3, CD4, CD19, CD8, HLA-DR, CD56, CD105, CD2, CD1c, CD25, CD49e, ROR1, CD209, CD9, SSEA4, HLA-BC, CD63, CD40, CD62P, CD11c, CD81, MCSP1, CD146, CD41b, CD42a, CD24, CD86, CD44, CD326, CD133/1, CD29, CD69, CD142, CD45, CD31, CD20, and CD14) plus two isotype controls (REA and IgG1).

The MACSPlex Exosome Detection Reagents for CD9, CD81, and CD63 were used to label the captured exosomes. Briefly, 6 µL and 80 µL of EVs from serum and urinary supernatant, respectively, were added to 114 µL and 40 µL of MACSPlex buffer, respectively, to obtain a final reaction volume of 120 µL. All samples were processed following the manufacturer’s instructions. One negative/blank control (MACSPlex Buffer only) was used in each run experiment to determine non-specific signals. To avoid non-specific signals, from the raw median fluorescence intensity (MFI) of each marker was subtracted the MFI of the negative/blank control used in the same run experiment. Values below the corresponding control were regarded as negative. The detection of FITC, PE, and APC fluorophores were measured for each sample. For each sample, the 39 bead populations (37 exosomal surface epitopes + 2 isotype controls) can be distinguished by different fluorescence intensities detected in the FITC, PE, and APC channels of a flow cytometer. Flow cytometry sample acquisition was carried out on a BD FACSCanto (Becton Dickinson, San Diego, CA, USA), equipped with two lasers, 488 nm and 630 nm, capable of detecting the necessary fluorescence signals. Data analysis was performed with the corresponding software (BD FACSDiva). 

### 2.6. Statistical Analysis

The aim of the study was to evaluate the technical feasibility based on a new workflow to investigate the potential role of EVs in early diagnosis of PCa. Due to the explorative nature of the study, no formal sample size calculations were performed.

Descriptive statistics were reported as proportions and median values (range). Non-parametric ranking test (Median test) was used to compare continuous data (age and PSA levels of patients). 

MACSPlex results were analyzed by one-way ANOVA followed by Tukey’s post hoc test correction for multiple comparisons.

To generate heatmaps, data were exported to comma-separated files, which were subsequently imported into R Software for further analysis and data visualization. 

All *p*-values were based on two-sided testing, and *p*-values < 0.05 were considered statistically significant. 

Statistical analysis was carried out using SAS software, version 9.4 (SAS Institute, Cary, NC, USA), GraphPad Prism 7 Software (GraphPadPrism Software, San Diego, CA, USA) and R statistical package version v 4.0.0 (R Foundation for Statistical Computing, Vienna, Austria).

## 3. Results

A summary of the case series and clinical data is reported in Supplementary Table S1. The PSA level was statistically significant to distinguish PCa patients vs. other categories (*p* = 0.007). 

**Figure 1:**
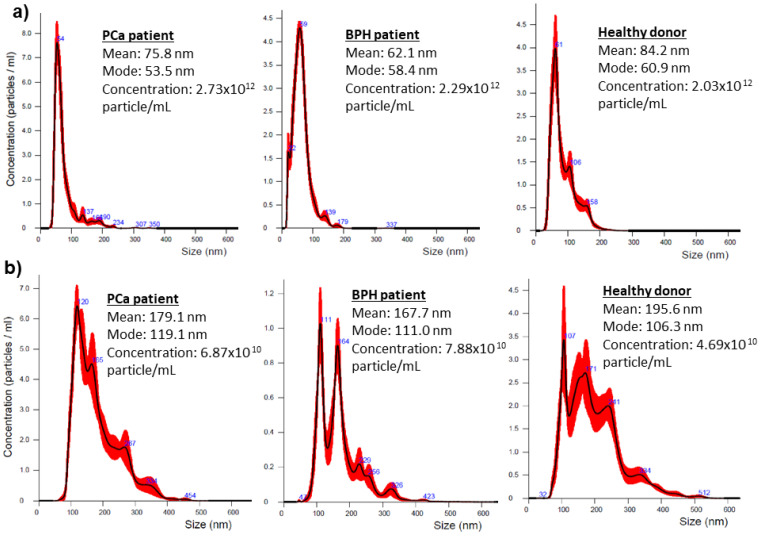
Representative distribution plot of serum (**a**) and urinary supernatant (**b**) EVs and corresponding concentration by Nanosight Instrument in the three groups.

EVs were successfully isolated from all serum and urinary supernatant samples. In agreement with recent literature [17,18,19], NTA analysis revealed a concentration of serum EVs in the range of ~10^12^ particle/mL, and of urinary EVs in the range of ~10^10^ particle/mL; the latter being slightly higher in size and endowed with wider heterogeneous size distribution than serum EVs (Figure 1). No statistical differences were found between the EV concentration among the three groups (serum EVs: *p* = 0.2851; urinary supernatant EVs: *p* = 0.3405). 

**Figure 2:**
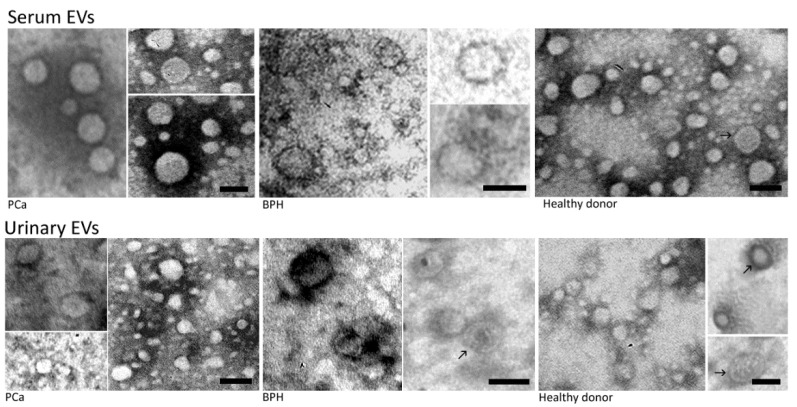
Negative staining of serum and urinary supernatant EVs from the same matched samples. TEM observations showed numerous EVs between 20 and 100 nm. Bar scale = 100 nm.

TEM analysis of serum and urinary EVs conveyed the presence of heterogeneous sets of 20 to 100 nm spherical-shaped structures with well-preserved membranes (Figure 2). 

**Figure 3:**
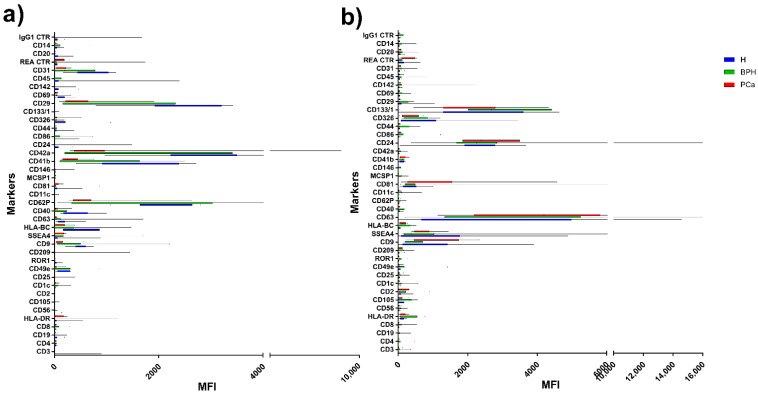
Range to the min and max of MFI for each EVs marker from serum (**a**) and urinary supernatant (**b**). MFI of healthy donors in blue; BPH patients in green; PCa patients in red.

**Figure 4:**
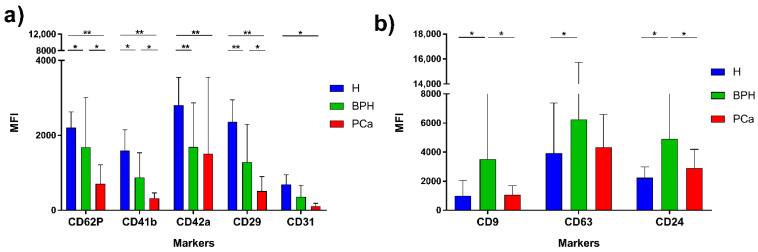
Range of the MFI of the significant markers found in serum EVs (**a**) and urinary EVs (**b**). Healthy donors in blue; BPH patients in green; PCa patients in red. * *p* < 0.05; ** *p* < 0.0001.

Flow cytometric analysis of EVs was then performed by the MACSPlex kit. The MFI of each marker is reported in Figure 3 and in Supplementary Tables S2 and S3. In keeping with previous literature [17,18,19], marker expression profile of serum and urinary supernatant EVs were markedly different. In particular, high expression of CD24 and CD133 was detected in urine EVs, the two markers being nearly absent in serum EVs [17]. MACSPlex analysis of serum EV in the three groups showed differences in the expression of five markers, namely CD62P, CD41b, CD42a, CD29, and CD31 (Figure 4). In particular, the expression levels of CD62P, CD41b, and CD29 were significantly different in PCa compared to BPH and H groups; CD42a expression level was higher in PCa and BPH vs. H; and CD31 was significantly different between PCa and H (Table 1). Regarding the urinary supernatant EVs, the MACSPlex results highlighted three significant markers (CD9, CD63, CD24) (Figure 4). In particular, CD9 and CD24 were different between BPH vs. PCa and H; whereas CD63 was significantly different between H and BPH (Table 2). 

The heatmap of serum EVs highlighted two main groups (the first compounds mostly of PCa and BPH patients and the second of H) characterized by different expressions of CD29, CD41b, CD62P, CD42a, and CD31 (Supplementary Figure S1). Similarly, the heatmap of urinary EVs showed a weak clustering associated with the different expressions of CD24, CD9, and CD63 among the groups (Supplementary Figure S2).

## 4. Discussion

PCa is a multifaceted disease, featuring several subtypes and clinical appearances. So far, a lot has been done to identify novel biomarkers and develop rapidly translatable assays for early detection, to discriminate between fast and slow-growing disease, and to predict an outcome. However, although several tests have been assessed, and some even become recently available, the unmet clinical need for novel biomarkers that can demonstrate improvement in these areas is still unsolved. Nowadays, the most utilized prostate marker for the detection of PCa is the debatable serum-based marker PSA. Despite its application, this test has low accuracy, is not specific enough, and can prompt unnecessary biopsies. A positive PSA result for PCa still needs confirmation through a tissue biopsy. Moreover, despite PSA monitoring and histopathological examinations being the gold standards in PCa diagnostics, they are not well suited for patient stratification, and predicting and monitoring treatment response. Thus, alternative diagnostic approaches are needed. Liquid biopsies have come to the rescue with the promise to find surrogate biomarkers that are easily detectable and feasible, and clinically useful, offering a unique chance to isolate tumor-derived material for clinical assessment. LB materials have been heavily investigated as minimally invasive tests to help oncologists to evaluate PCa patients with real-time cellular or molecular information [20,21,22,23]. In this context, urine is a likely source for the detection of PCa biomarkers. It is a less complex and relatively clean fluid in respect to whole blood and can be even enriched in biomarkers after manipulation of the prostate [24]. EVs transport several different kinds of molecules to deliver messages to target cells, mainly bioactive molecules such as nucleic acids, proteins, and lipids, in paracrine and/or endocrine ways. EVs can guide local and systemic intercellular communications, remodeling the normal and tumor microenvironment, potentially regulating cancer metabolism, with the capability to induce drug resistance, angiogenesis, and metastasis [25,26,27]. They have been shown to be potentially diagnostic and prognostic markers suggesting their role in precision medicine and disease management [11,28].

Since the tumor is heterogeneous, it is easy to understand that a single biomarker cannot fully reflect or monitor the disease or its stage, or follow its progressions. Evaluating more than single markers at one time can be fruitful. However, presently available EV analysis methods are expensive and time-consuming. The development of a fast, easy-to-perform, and efficient method for detecting multiple proteins in a single reaction could be worth undertaking [29]. Combining these elements, urine results in an interesting starting material to detect and characterize disease related-EVs. Herein, in a small pilot case series, we performed a small feasibility study to compare urine and serum as a starting material for the detection and characterization of EVs isolated by a resin-based approach. We evaluated the feasibility and the advantages of a novel workflow easily translatable into the clinic for the enrichment and characterization of EVs, enabling a LB test for the non-invasive profiling of multiple exosomal antigens. Despite the main study’s limitation, which is the fact that is a small case series, our results showed that it is possible to detect EVs populations in these settings, clearly confirming that studying nanoparticles in both urine and serum for PCa investigation is feasible and translatable into the clinic. Interestingly, probably due to the nature of the starting material, results between urine were not completely comparable showing the expression of different antigens. The MACSPlex-based characterization, in fact, showed that the antigens differentially expressed in the groups we investigated were dissimilar between serum (CD62P, CD41b, CD42a, CD29, and CD31) and urine supernatants (CD9, CD63, and CD24), firstly suggesting the different origins of EVs. Then, regarding the antigens detected in the serum, these are potentially derived from EVs related to endothelial cells and platelets. Since endothelial cells and platelets could have a key role in tumor growth, metastasis, and cancer-associated thrombosis, the detection of EVs presenting these antigens could a have a possible use, e.g., in revealing and monitoring cancer-related venous thromboembolism, a circumstance associated with increased morbidity and mortality [30,31]. Indeed, the role and involvement of EVs in cancer-related hypercoagulation is worthy of being investigated deeply, as already suggested [32]. Moreover, recent studies showed that, in human serum, most EVs are platelet-derived [33,34,35], playing a role in several physiological but also pathological conditions such as inflammation [36] and tumor progression [37]. Interestingly, studies characterizing urinary-derived vesicles, identified EVs derived from cell-free urine of PCa patients CD63, CD24 and CD9 positive, which is in agreement with our results, but dimensionally below 100 nm [38]. Moreover, we found urinary EVs positive for CD133 and CD24, as previously reported [39]. In particular, CD24 is overexpressed in many cancers and appears to be oncogenic [40]. It has been already demonstrated to have a key role in maintaining urothelial cancer stem-like traits and to be a potential urinary biomarker for the carcinoma of the bladder [41]. Since a long time, its overexpression has been significantly associated with a more aggressive course of a number of neoplastic diseases, and it is increasingly used as a marker to identify tumor-derived EVs in body fluids for diagnostic purposes [42,43]. Again, our data demonstrated the detection of urine-derived-EV antigens related to cancer aggressiveness and that progression is feasible and a worthy option to be further evaluated.

On the other hand, it is interesting to note that the vesicle markers altered in BPH and cancer patients were somewhat expressed at lower levels in respect to those detected in healthy donors. To our knowledge, this is quite intriguing since it is generally believed that a pathological condition should be associated with an increase of one or more markers. In literature, there are cases of vesicle marker decrease, not only in terms of EVs-related antigens and not only in cancer setting. For example, in papillary thyroid carcinoma serum exosomal miR-29a, expression levels were significantly decreased in respect to healthy controls [44]. In hypertension, specific exosomal miRNAs have been shown to be downregulated, in particular after specific stresses such as TGF-β stress [45]. Moreover, it is known that growth factors can induce regulatory effects on exosome release [46]. Indeed, Zhou et al. observed the role of EGFR on exosome production, showing that the production was significantly decreased in cells treated with this factor [47]. Hence, it is conceivable that certain pathophysiological conditions might reduce EVs concentration, epitopes, and loads. In agreement, e.g., during nephrotic syndrome, the decreased release of exosomal marker proteins into the urine was observed also [48]. Hence, although it is necessary to consider these result carefully, and knowing they have to be validated in larger patient cohorts, they could suggest that a specific state could be paralleled by a decrease of certain vesicle populations. 

Finally, our data that is in agreement with literature suggest that to simultaneously analyze both kinds of materials could offer a wider point of view on PCa, being more informative and potentially useful towards a diagnostic intent [49]. Moreover, our method is fast, easily performable, and quite cost effective. The detection of multiple antigens thanks to a phenotyping approach is already extremely useful in hematological malignancies and translating this possibility to a solid tumor could be of enormous importance. Hence, despite the fact that a greater case series is naturally needed, our study suggests that liquid biomarkers, and herein EVs presenting different antigens in urine and serum, could be worthy of being investigated to deepen the role of urine markers in PCa or other urological cancer. In the next future, it could be possible to gain information regarding prognosis, treatment response, and treatment selection [22].

## Figures and Tables

**Table 1:** A summary of the significant statistical results for serum EVs using one-way ANOVA test.

Tukey’s Multiple Comparisons Test	Mean Diff	95% CI	Adjusted *p*-Value
CD62P			
Healthy vs. BPH	521.9	31.84 to 1012	0.0336
Healthy vs. PCa	1497	1007 to 1987	<0.0001
BPH vs. PCa	975.1	485 to 1465	<0.0001
CD41b			
Healthy vs. BPH	723.7	233.6 to 1214	0.0016
Healthy vs. PCa	1275	784.5 to 1765	<0.0001
BPH vs. PCa	550.9	60.84 to 1041	0.023
CD42a			
Healthy vs. BPH	1120	630.2 to 1610	<0.0001
Healthy vs. PCa	1300	809.6 to 1790	<0.0001
CD29			
Healthy vs. BPH	1073	582.4 to 1563	<0.0001
Healthy vs. PCa	1848	1358 to 2338	<0.0001
BPH vs. PCa	775.7	285.6 to 1266	0.0006
CD31			
Healthy vs. PCa	585.4	95.34 to 1075	0.0142

**Table 2:** A summary of the significant statistical results for urinary supernatant EVs using one-way ANOVA test.

Tukey’s Multiple Comparisons Test	Mean	95% CI	Adjusted *p*-Value
CD9			
Healthy vs. BPH	−2532	−4398 to −665.4	0.0043
BPH vs. PCa	2450	482.5 to 4417	0.0099
CD63			
Healthy vs. BPH	−2315	−4181 to −448.7	0.0103
CD24			
Healthy vs. BPH	−2668	−4534 to −802.1	0.0024
BPH vs. PCa	1997	29.58 to 3964	0.0457

## Data Availability

The data presented in this study are available in Supplementary Tables S2 and S3.

## Supplementary Materials

The following are available online at https://www.mdpi.com/2075-4418/11/3/466/s1. Figure S1: Heatmap analysis of MFI of each serum EVs markers, Figure S2: Heatmap analysis of MFI of each urinary EVs markers, Table S1: Prostate cancer case series summary, Table S2: Median and mean values of fluorescence intensity of each marker in all serum EVs samples. Table S3: Median and mean values of fluorescence intensity of each marker in all urinary supernatant EVs samples.
