# Supplementary material for: Detection and Investigation of Extracellular Vesicles in Serum and Urine Supernatant of Prostate Cancer Patients
Source: Diagnostics (Basel). 2021 Mar 8;11(3):466. doi: 10.3390/diagnostics11030466 (PMC7998238; doi:10.3390/diagnostics11030466)
Supplement: Supplementary file 1 [file diagnostics-11-00466-s001.pdf]

Suppl. Figure S1. Heatmap analysis of MFI of each serum EVs markers.

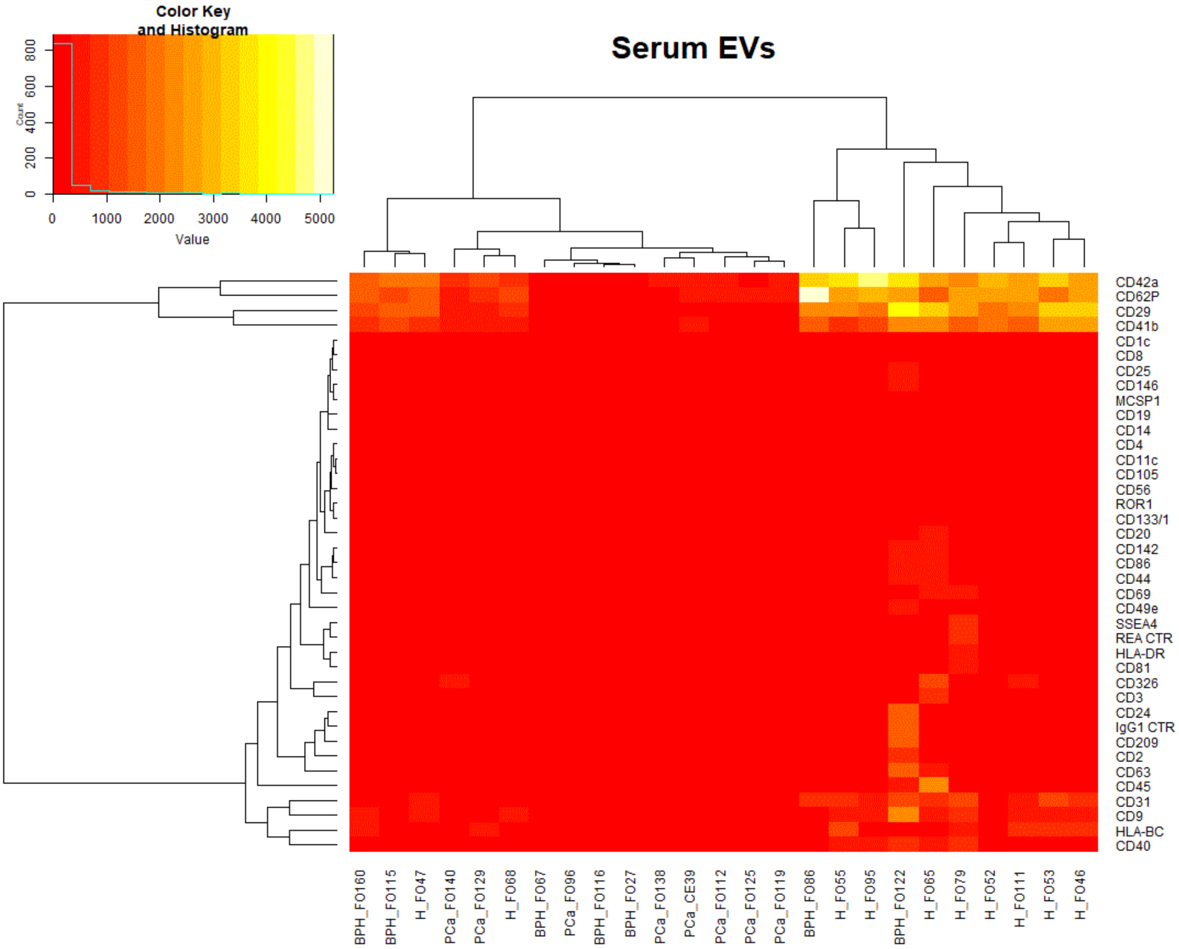

Suppl. figure S2. Heatmap analysis of MFI of each urinary EVs markers.

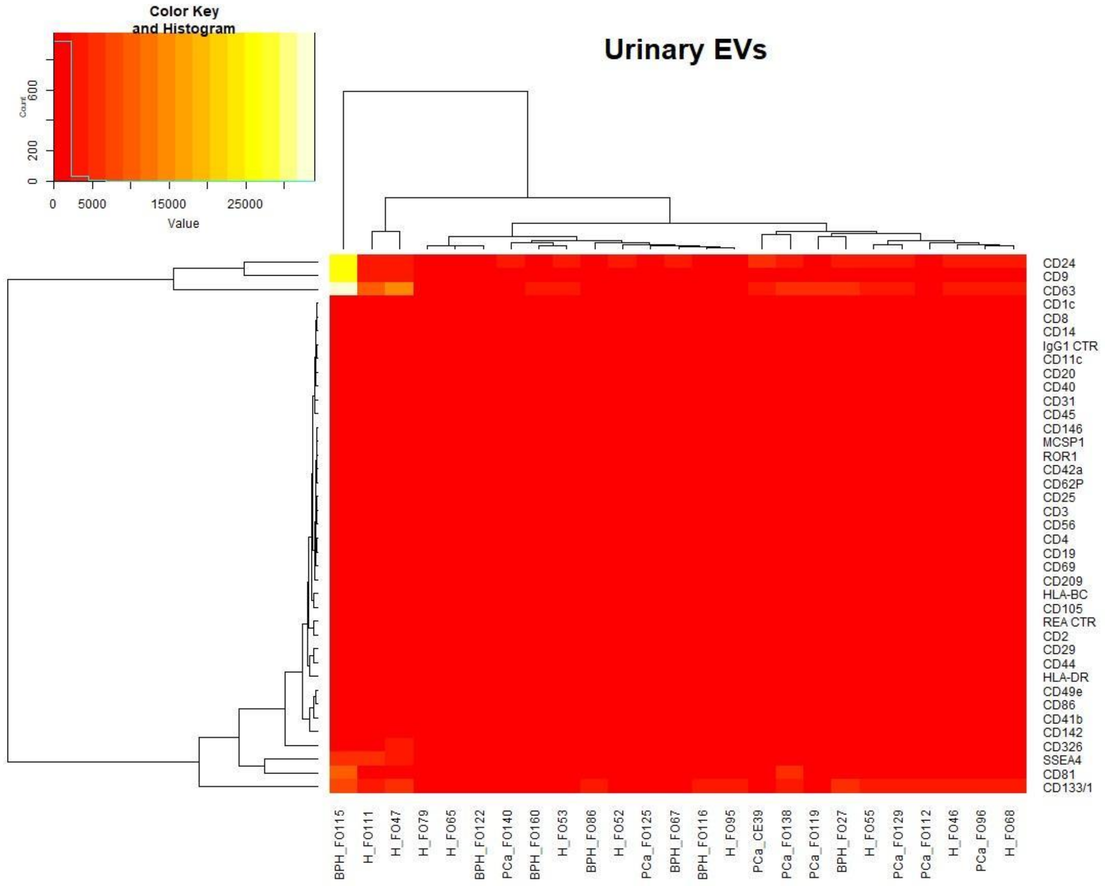

Suppl. Table S1. Prostate cancer case series summary.

	PCa	BPH	Healthy donors	*p-value*
**n**	10	10	10	
**Median age, y (range)**	73 (68-83)	74 (61-83)	53 (50-80)	0.067
**Median baseline PSA level, ng/mL (range)**	8.78 (0.49-118.40)	5.18 (1.38-11.00)	0.92 (0.59-5.29)	0.007
**Gleason score**		-	-	-
**≤6**	3			
**>6**	7			
**Stage**		-	-	-
**pT2**	5			
**pT3**	2			
**Unknown**	3			

PCa: prostate cancer patients; BPH: benign prostatic hyperplasia patients; PSA: prostate specific antigen

lementary Table S2: Median and mean values of fluorescence intensity of each marker in all serum EVs samples.

	Healthy donors		BPH		PCa	
	Median value	Mean value	Median value	Mean value	Median value	Mean value
**CD3**	0	89.8	0	0	0	0
**CD4**	10.5	15	14	41.5	12.5	14.6
**CD19**	0	34.5	0	24.8	0	9.7
**CD8**	11	12.2	7.5	59.6	6.5	12.6
**HLA-DR**	5.5	67.3	6.5	126.8	13.5	69.4
**CD56**	0	13.1	0	5.5	0	0
**CD105**	0	0	0	9.4	0	0
**CD2**	0	0.1	0	97.3	0	0.9
**CD1c**	8.5	11.3	12	59.4	7.5	19
**CD25**	0	0	0	38.7	0	0
**CD49e**	190.5	175.3	10	177.4	3.5	34.1
**ROR1**	9.5	26.9	0	7.1	0	5.1
**CD209**	0	0	0	144.5	0	0
**CD9**	471.5	496.6	188.5	413.6	59.5	121.4
**SSEA4**	7	105.2	6	219.5	18.5	69.5
**HLA-BC**	478	509.2	131.5	281.7	69.5	115.4
**CD63**	114	166.5	43.5	215.1	22.5	21.9
**CD40**	233	390.4	78	167.6	25	60.3
**CD62P**	2505	2205.8	922	1683.9	462.5	708.8
**CD11c**	0	0	0	10.8	0	0.4
**CD81**	17.5	70.5	0	94.8	0.5	40.6
**MCSP1**	0	12.7	3	36.3	15	12
**CD146**	3.5	19.9	4	44.2	0	9.6
**CD41b**	1601.5	1597.2	477	873.5	251.5	322.6

**CD42a**	2664	2810.4	1286	1690.1	602	1510.7
**CD24**	14.5	62.4	0	150	0	2.2
**CD86**	0	48.3	0	115.7	0	0
**CD44**	16	52.2	0	49.8	0	6.7
**CD326**	27.5	183.1	0	30.4	0	63.2
**CD133/1**	0	12.3	0	8.7	0	0
**CD29**	2281	2364.4	771.5	1291.9	280	516.2
**CD69**	79	135.2	32.5	62.4	25	32
**CD142**	11.5	68.1	0	40.8	0	0
**CD45**	34	268	0	140.5	0	0.8
**CD31**	647.5	696.1	194.5	361.6	66	110.7
**REA CTR**	2	95	14	188.8	25	91.7
**CD20**	30	65.3	0	4.4	0	2.4
**CD14**	26	35.3	21.5	105.5	0	12.8
**IgG1 CTR**	4.5	33.3	0	167.7	0	1.3

Supplementary Table S3: Median and mean values of fluorescence intensity of each marker in all urinary supernatant EVs samples.

	**Healthy donors**		**BPH**		**PCa**	
	**Median value**	**Mean value**	**Median value**	**Mean value**	**Median value**	**Mean value**
**CD3**	0	7.3	7	60.25	1	22.375
**CD4**	3	4.5	5.5	76	4.5	9
**CD19**	0	0	0	56.875	0	2.75
**CD8**	10.5	10.6	15	90.5	5.5	9.875
**HLA-DR**	88.5	108.9	71	240.875	94.5	129.125
**CD56**	3	17	13	51	22.5	26.625
**CD105**	39.5	76.3	38	156.875	52.5	79.625
**CD2**	37.5	79.3	82	192.375	103	146.5
**CD1c**	0	8.4	3.5	89.875	4.5	12.875
**CD25**	1	14.5	4.5	54.875	1	25.875
**CD49e**	8.5	25.3	29.5	226.625	9.5	30.75
**ROR1**	11	13.5	32	70	20	15.25
**CD209**	14	31.6	22	93.75	35.5	61.625
**CD9**	346.5	994	252	3525.5	1038.5	1076
**SSEA4**	429.5	1117.3	375	1173.875	619	724.625
**HLA-BC**	32.5	65.8	25	137.25	130.5	130.75
**CD63**	2753.5	3934.8	2062.5	6249.625	4197.5	4334.5
**CD40**	12	30.9	8.5	114.625	23	25.125
**CD62P**	2	7.9	10.5	45.875	2	13.5
**CD11c**	4.5	8.6	23	115.625	3	11.75
**CD81**	367	395.7	339	1403.375	639	1184.125
**MCSP1**	0	1.1	5	54.75	2.5	6
**CD146**	1.5	5.7	5	57.75	0.5	6.375

**CD41b**	96.5	102.8	117	239.375	94.5	127.125
**CD42a**	5	9.3	12.5	53.125	23.5	27.875
**CD24**	2466	2242.7	2258.5	4910.875	2388	2914.25
**CD86**	8	16	65.5	207.25	48.5	43.75
**CD44**	24.5	79.3	26	161.25	37	37.875
**CD326**	327.5	715.5	253	436.5	319.5	345.125
**CD133/1**	2483	2331	2683	3269.25	2151.5	2148.375
**CD29**	20.5	136	31	130.625	41.5	38.875
**CD69**	19	26.5	18	76.5	13	16.625
**CD142**	17.5	28.7	34	314.25	47	47.5
**CD45**	7	24.9	19	134.375	19.5	36
**CD31**	15.5	36.6	50.5	109.5	49.5	56.5
**REA CTR**	28.5	120.1	62.5	169	192.5	252
**CD20**	17	40.3	38	117.5	56	50.875
**CD14**	23	21.9	54.5	109.5	7.5	19.125
**IgG1 CTR**	8	18.9	16.5	119.25	21.5	24.125
